# Dynamic FDG PET Imaging to Probe for Cardiac Metabolic Remodeling in Adults Born Premature

**DOI:** 10.3390/jcm10061301

**Published:** 2021-03-22

**Authors:** Philip A. Corrado, Gregory P. Barton, Francheska C. Razalan-Krause, Christopher J. François, Naomi C. Chesler, Oliver Wieben, Marlowe Eldridge, Alan B. McMillan, Kara N. Goss

**Affiliations:** 1 Department of Medical Physics, University of Wisconsin-Madison, Madison, WI 53705, USA; pcorrado2@wisc.edu (P.A.C.); gregory.barton@utsouthwestern.edu (G.P.B.); owieben@wisc.edu (O.W.); abmcmillan@wisc.edu (A.B.M.); 2 Department of Internal Medicine, University of Texas Southwestern Medical Center, Dallas, TX 75390, USA; 3 Department of Pediatrics, University of Wisconsin-Madison, Madison, WI 53705, USA; meldridge@pediatrics.wisc.edu; 4 Department of Medicine, University of Wisconsin-Madison, Madison, WI 53705, USA; razalan@wisc.edu; 5 Department of Radiology, University of Wisconsin-Madison, Madison, WI 53705, USA; Francois.Christopher@Mayo.edu; 6 Edwards Lifesciences Center for Advanced Cardiovascular Technology, University of California, Irvine, CA 92697, USA; nchesler@uci.edu; 7 Department of Biomedical Engineering, University of California, Irvine, CA 92697, USA

**Keywords:** positron emission tomography (PET), fluorodeoxyglucose (FDG), glucose, cardiac metabolism, premature birth

## Abstract

Individuals born very premature have an increased cardiometabolic and heart failure risk. While the structural differences of the preterm heart are now well-described, metabolic insights into the physiologic mechanisms underpinning this risk are needed. Here, we used dynamic fluorodeoxyglucose (FDG) positron emission tomography/magnetic resonance imaging (PET-MRI) in young adults born term and preterm during normoxic (N = 28 preterm; 18 term) and hypoxic exposure (12% O_2_; N = 26 preterm; 17 term) to measure the myocardial metabolic rate of glucose (MMRglc) in young adults born term (N = 18) and preterm (N = 32), hypothesizing that young adults born preterm would have higher rates of MMRglc under normoxic conditions and a reduced ability to augment glucose metabolism under hypoxic conditions. MMRglc was calculated from the myocardial and blood pool time-activity curves by fitting the measured activities to the 3-compartment model of FDG kinetics. MMRglc was similar at rest between term and preterm subjects, and decreased during hypoxia exposure in both groups (*p* = 0.02 for MMRglc hypoxia effect). There were no differences observed between groups in the metabolic response to hypoxia, either globally (serum glucose and lactate measures) or within the myocardium. Thus, we did not find evidence of altered myocardial metabolism in the otherwise healthy preterm-born adult. However, whether subtle changes in myocardial metabolism may preceed or predict heart failure in this population remains to be determined.

## 1. Introduction

Preterm births account for roughly 10% of all live births globally [1]. Recent advances in the treatment of extremely premature infants have led to decreases in mortality [2], such that the long-term ramifications of premature birth are only now becoming apparent. A recent Swedish registry study of individuals born in the past 2 decades identified a 17-fold increased risk of developing heart failure by young adulthood in individuals born extremely premature (gestational age <28 weeks) [3]. Premature birth is also associated with changes in cardiac morphology, including smaller ventricular chamber sizes and reduced stroke volume index (SVi) [4,5,6]. Functionally, left ventricular ejection fraction is preserved, though some studies report slightly reduced right ventricular (RV) ejection fraction (EF) [6,7]. Additional studies demonstrate impaired cardiac reserve during exercise in adults born premature, including an inability to fully augment stroke volume during submaximal exercise [8,9] and a slower heart rate recovery after exercise [10]. Despite these findings, metabolic insights into the physiologic mechanisms which may play a role in increased cardiometabolic risk in individuals born premature are lacking.

Prior studies in high-risk non-preterm cardiac populations demonstrate that cardiac metabolic shifts may precede structural or functional changes. For example, in patients with left ventricular hypertrophy, changes in cardiac contractile reserve are frequently preceded by shifts away from fatty acid oxidation to an increased preference for glucose metabolism [11]. Prior studies in patients with RV hypertrophy and dysfunction secondary to pulmonary hypertension demonstrate that glucose uptake by positron emission tomography (PET) imaging correlates with the degree of RV dysfunction [12] and with reduced survival [13]. In addition, the fetal myocardium is highly glucose avid, transitioning to fatty acid metabolism post-birth, and impaired cardiopulmonary transition is generally associated with worse neonatal morbidity [14,15,16]. However, whether early shifts in cardiac metabolism are present in adults born preterm remains unknown. 

(18)F-fluorodeoxyglucose (FDG) PET can probe myocardial glucose metabolism, providing a potential non-invasive early marker of cardiac disease. In addition, hypoxia exposure has the ability to exacerbate differences in cardiac glucose metabolism. Cardiac glucose utilization may increase acutely as much as 70% during hypoxia exposure in humans, despite unchanged glucose uptake in other tissues [17]. Recently, dynamic imaging using a constant, slow ^18^F-FDG infusion has been utilized to capture metabolic responses to stressors such as hypoxia [18,19]. Specific to cardiac metabolism, acute intra-scanner hypoxia exposure can acutely alter myocardial glucose metabolism in pigs [20], representing a novel method to capture dynamic cardiac metabolic substrate flexibility.

In this study, we used dynamic FDG PET-MRI before and during hypoxia exposure to measure the myocardial metabolic rate of glucose (MMRglc) in young adults born premature. The skeletal metabolic rate of glucose (SkMMglc) was also assessed as a comparator. We aimed to assess differences in glucose utilization and substrate flexibility as a potential mechanism of impaired cardiac function and reserve that may contribute to the increased risk of heart failure in this population. Based on their overall increased cardiometabolic and heart failure risk, our hypothesis was that young adults born preterm would have higher rates of glucose metabolism under normoxic conditions and a reduced ability to augment glucose metabolism under hypoxic conditions as compared to control subjects.

## 2. Materials and Methods

### 2.1. Participants and Study Design

Young adult participants born moderately to extremely premature (N = 32) were recruited and allocated to the study intervention from the Newborn Lung Project, a cohort of infants born with very low birth weight (≤1500 g) between 1988 and 1991 in Wisconsin and Iowa and followed prospectively at the University of Wisconsin-Madison [21,22], or from the general public with confirmation of birth history from neonatal records (birth weight ≤1500 g or gestational age ≤32 weeks). As such, all preterm subjects met one of the following criteria: birth weight ≤1500 g or gestational age ≤32 weeks. Age-matched term-born participants (N = 18) were recruited from the local population through internet posting at the university job center or Craigslist, by a mass university-wide email, and via flyers announcing the study posted at the university and university clinics. Interested individuals then contacted the study coordinator to enroll. All subjects were free of known cardiac or metabolic disorders with normal fasting blood glucose. The participants were informed of the purpose and risks of the study and provided written informed consent in accordance with the standards set by the Declaration of Helsinki. The protocols were approved by the Institutional Review Board at the University of Wisconsin-Madison (Application ID: 2017-0238). The trial was registered with the U.S. National Library of Medicine (ClinicalTrials.gov ID: NCT03245723). 

In order to minimize the acute nutritional effects on cardiac metabolism, eligible subjects were asked to provide oral consent as part of phone screening for a pre-visit fast and low-carbohydrate, high-protein diet. The following instructions were provided: A complete fast is required overnight (or a minimum of 8 h) prior to arrival for the study visit, including candy and gum. Only water is permitted; flavored water is not allowed. No caffeine, alcohol, or nicotine for 12 h prior to the scan. A high-protein, low-carbohydrate diet the day prior to imaging was strongly encouraged, but no specific meal plan was prescribed. Upon arrival, the subjects were asked whether they completed the 12-h fast or the prior-day high-protein, low-carbohydrate diet. The subjects also completed a resting electrocardiogram (ECG) upon arrival to confirm the absence of cardiac arrhythmias prior to imaging and acute hypoxia exposure. Prior to PET imaging, all participants underwent height and weight measurements and physical activity level assessment via the global physical activity questionnaire [23]. Finally, the subjects underwent PET scanning using a rest-stress (normoxic-hypoxic) protocol. 

### 2.2. PET/MRI Image Acquisition

**Figure 1:**
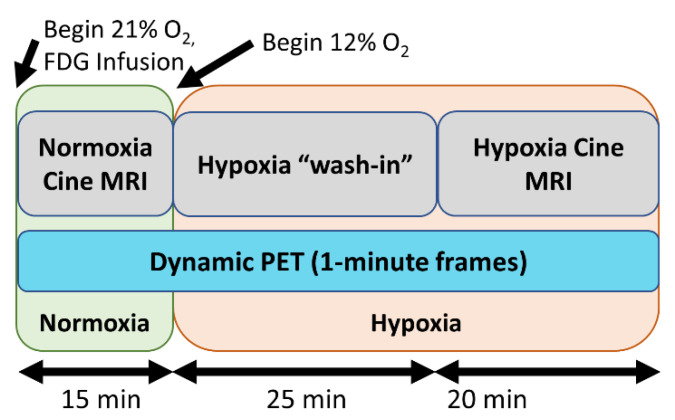
Imaging protocol: In the first 15 min, the subjects breathe 21% oxygen. Subsequently, the oxygen levels are reduced to 12% for 45 min. Cine MRI data are acaquired during the normoxia and, after a 25 min period of a hypoxia ‘washin’ phase, during hypoxia. (18)F-fluorodeoxyglucose (FDG) is continuously infused and dynamic PET data are continuously acquired throughout the imaging exam.

The participants underwent simultaneous cardiac PET/MRI scanning on a 3.0 T scanner (GE Signa PET/MRI Discovery 750W, GE Healthcare, Waukesha, WI, USA) acquired while breathing normoxic followed by hypoxic gas. Cardiac MRI sequences were obtained as previously described [6]. Coincident with the onset of PET scanning, FDG was infused into the right antecubital vein at a constant rate of 0.01 mL/s for 60 min using a power injector, with an initial FDG activity of 10 mCi. To account for the volume of the tubing, a small bolus of 8 mL was delivered prior to continuous infusion to ensure that the line contained radiotracer and that there was a detectable blood FDG signal. Subjects initially breathed room air gas (21% O_2_) through a face mask with a two-way valve attached to a hose connected to a normoxic air bag for the first 15 min of the scan to establish resting glucose uptake. The hose was then switched to a hypoxic gas bag, and subjects then breathed hypoxic gas (12% O_2_) for the remaining 45 min (total PET duration and FDG infusion of approximately 60 min) to establish cardiac glucose utilization during hypoxia (Figure 1). Serum glucose and lactate levels were drawn from an antecubital vein every 10 min during the scan. Oxygen saturations and heart rate were monitored throughout the study. Reconstruction parameters for dynamic PET were as follows: 60-s frame duration, 45 cm FOV, matrix 192 × 192, 28 subsets, 2 iterations, VPFX-S, time-of-flight, and 5 mm smoothing. The MRI protocol acquired cardiac-gated short-axis cine images during breath holds with the following parameters: 35 × 35 cm^2^ FOV, 13-s breath holds, 1.4 × 1.4 mm^2^ spatial resolution, 7 cm slice thickness, and 20 acquired/reconstructed cardiac time frames. These MRI images covered the entire LV and RV.

### 2.3. Image Analysis

**Figure 2:**
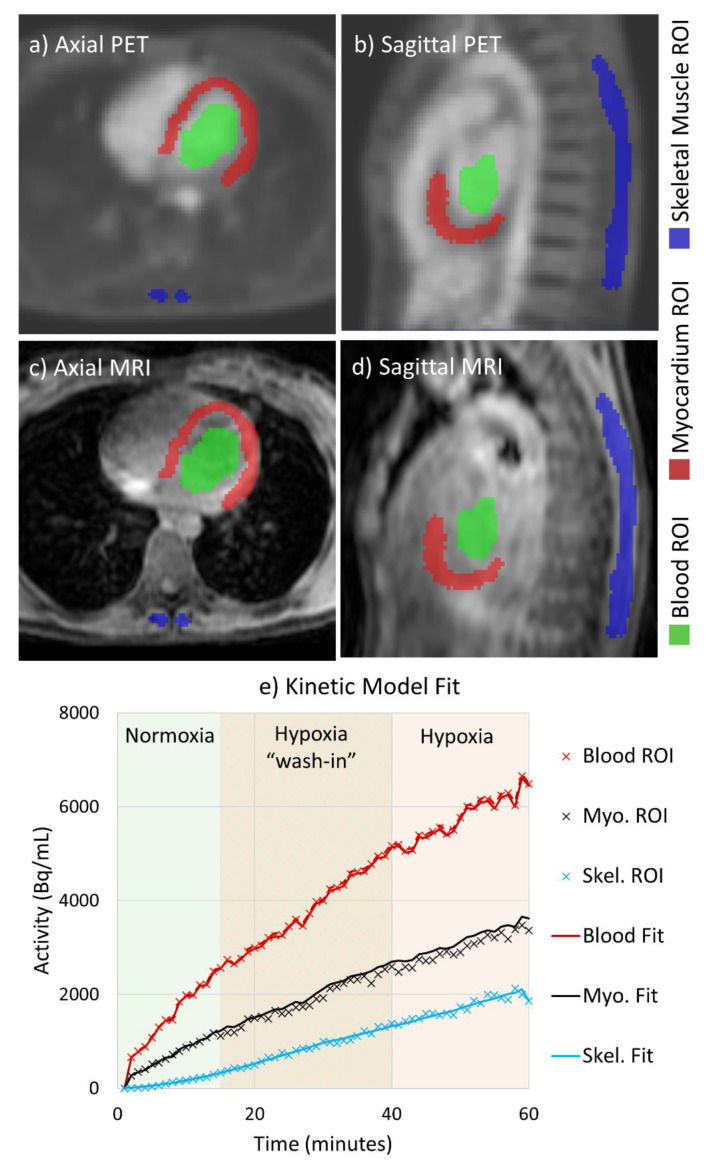
PET image processing method. Regions of interest (ROIs) were draw on the left ventricular (LV) myocardium, the LV cavity, and the paraspinal muscles using temporally averaged PET images (**a**,**b**) and co-registered MRI images (**c**,**d**). (**e**) the measured time-activity curves for each ROI (x’s on the graph) were used to fit 3-compartment models of FDG in the myocardium and skeletal muscle, in order to calculate the myocardial metabolic rate of glucose (MMRglc) and skeletal muscle metabolic rate of glucose (SkMRglc). The amount of F-18 fitted to each compartment over time is plotted as solid lines.

MRI measurements were made by our institution’s imaging core analysis lab and were previously reported [6]. Using temporally averaged PET images and co-registered MR images used for attenuation correction, three dimensional regions of interest (ROIs) were manually drawn over LV myocardium (including the septum), the LV cavity (blood pool), and the paraspinal muscles in ITK-SNAP analysis software [24]. Right ventricular free wall myocardial uptake was not assessed due to the thin nature of the myocardial wall, essentially at the thickness level of the PET voxel. The ROIs were analyzed to assess relative FDG uptake in active (myocardial) and passive (skeletal) muscles. The ROIs were eroded by 3 voxels in order to limit partial volume effects, and each ROI was applied to dynamic PET images to extract average activity value for each time point and establish time-activity curves for each tissue. Example ROIs are shown in Figure 2. The myocardial metabolic rate of glucose (MMRglc) was calculated from the myocardial and blood pool time-activity curves by fitting the measured activities to the FDG 3-compartment model [25] while allowing for spillover between the myocardium and blood pool ROIs [26]. The model was fit in a home-built script written in MATLAB (The MathWorks Inc., Natick, MA, USA) utilizing non-linear least squares fitting (via the “fmincon” function). The spillover-corrected blood pool activity computed in the myocardium model fit was then used to fit a 3-compartment model of the FDG dynamics in the skeletal muscle to compute the skeletal muscle metabolic rate of glucose (SkMRglc). In the skeletal muscle model, spillover was allowed from the blood pool compartment to the skeletal muscle ROI but not from skeletal muscle compartment to the blood pool ROI. Lumped constant values of 1.44 [27] and 1.16 [28] were used for the myocardium and skeletal muscle, respectively.

### 2.4. Statistical Analysis

Our original target sample size was 66 subjects, arrived at based on values from a swine study using the same protocol [20], and estimating a 30% increase in normoxic resting glucose utilization in young adults born premature using a 2:1 enrollment strategy with a significance level of 0.05 and a power of 0.80. The 2:1 enrollment strategy favoring preterm subjects was chosen based on a power analysis to detect differences in cardiac glucose metabolism and cardiac function using PET/MRI, with the hope to evaluate additional neonatal factors contributing to altered metabolism if the primary endpoint was met. The study was stopped early, before 66 subjects were enrolled, when in-person research was halted by our institution due to the COVID-19 pandemic.

Values are given as mean (standard deviation [SD]), and confidence intervals (CIs) are 95% CIs unless otherwise specified. All PET (MMRglc and SkMRglc), serum (glucose and lactate), vital sign (HR, SpO_2_, SBP, DBP, and RPP), and MRI (LV EDVi, LV ESVi, LV SVi, LV EF, CI) measures were analyzed using separate linear mixed-effects models with terms for birth status (term vs. preterm), gas (normoxic vs. hypoxic), and the interaction of gas and birth status. The difference in metabolic flexibility—or the ability to alter glucose metabolism in response to hypoxia—was computed as the hypoxia-preterm interaction coefficient in the MMRglc and SkMRglc models. For the measures taken every 10 min (serum levels and vital signs), the 55-min measurement is reported as the hypoxia measurement. Univariate linear regression was used to compare the change in SpO_2_ to the changes in MMRglc and SkMRglc with hypoxia, with separate regressions for the term and preterm groups. Significance levels were determined a priori at 0.05. Statistical analyses were performed using Prism Graphpad (Version 8, GraphPad Software Inc., La Jolla, CA, USA).

## 3. Results

### 3.1. Enrollment and Baseline Characteristics

**Figure 3:**
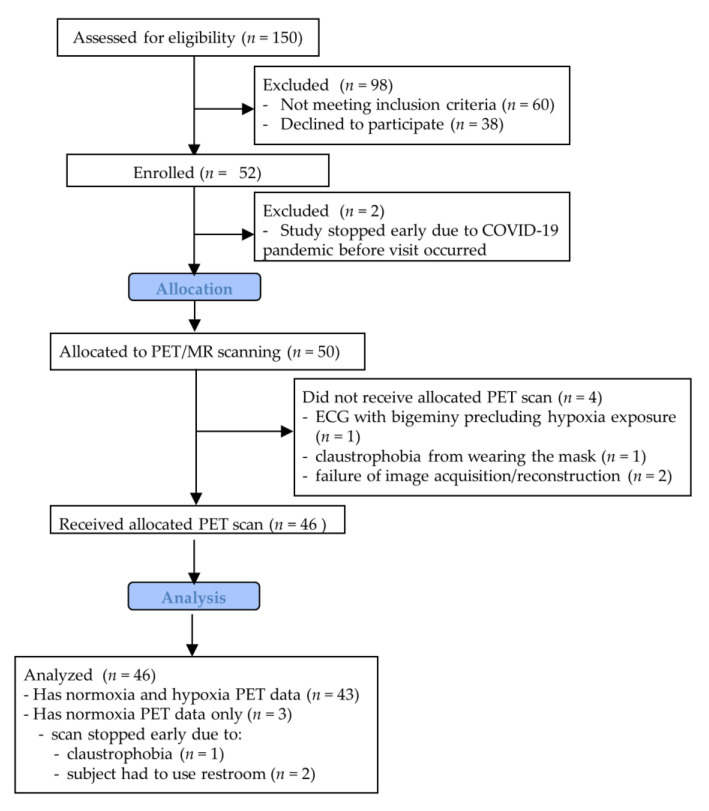
Schematic showing participant flow through the study.

Figure 3 shows a flow chart of participant enrollment in the study. This study has normoxia PET data for 46 subjects (28 preterm) and hypoxia PET data for 43 subjects (26 preterm). Table 1 shows the baseline characteristics and anthropomorphic measures for all subjects with any PET data available. Of the 46 subjects with normoxia PET data, all said they completed the 8-h fast prior to imaging and 43 of 46 (93%) said they followed a high-protein, low-carbohydrate diet the day before. The average gestational age for the preterm group was 29.2 weeks (range of 24–34 weeks, with birth weight range of 510–2360 g). The average gestational age for the term group was 39.8 weeks (range 37–42 weeks, with birth weight 2470–4540 g).

### 3.2. Vital Signs, Serum Glucose/Lactate, and Cardiac MRI Structure/Function

Table 2 shows normoxic and hypoxic measurements of vital signs and cardiac MRI structure and function. As reported in the companion MRI paper in this special issue (“Exaggerated Cardiac Contractile Response to Hypoxia in Adults Born Preterm”), preterm subjects had smaller left ventricles (*p* = 0.01 for EDVi, *p* = 0.001 for ESVi) that were hypercontractile (*p* = 0.01 for LV EF). In response to the hypoxic gas, both term and preterm subjects reduced ventricular volumes and increased cardiac index. Sp0_2_ fell gradually during the hypoxic gas portion of the study from averages of 98% and 98% in term and preterm subjects respectively to averages of 85% and 81% in term and preterm subjects by the 55-min mark. Conversely, heart rate rose gradually but similarly during the hypoxic gas portion of the study from averages of 67 and 69 beats per minute in term and preterm subjects to averages of 72 and 81 in term and preterm subjects by the 55-min mark.

**Figure 4:**
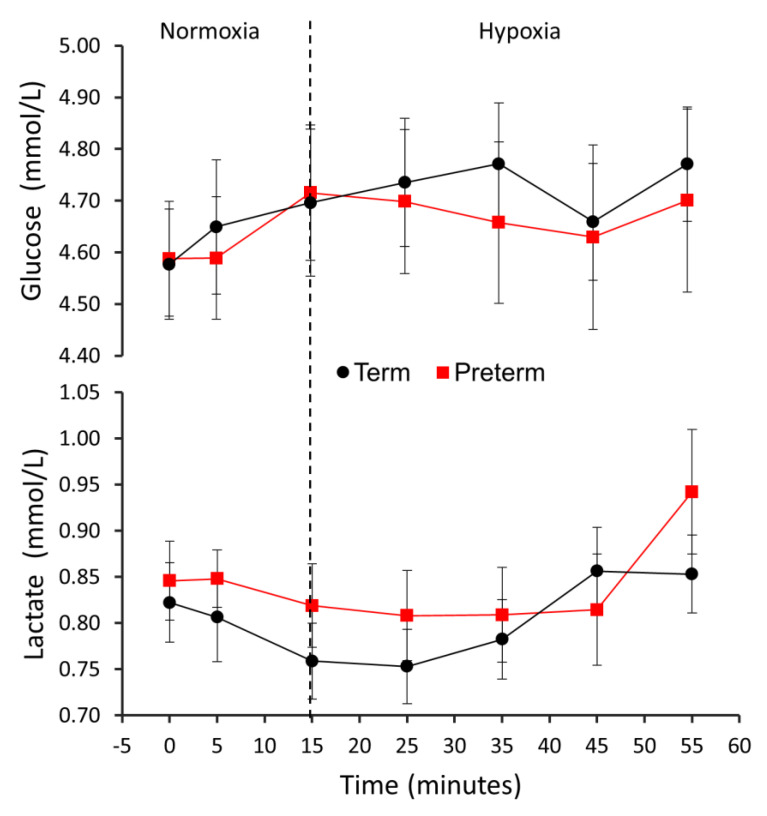
Serum glucose and lactate levels over the course of the PET experiment. The vertical dotted line represents the time when the oxygen level delivered to the subjects’ breathing masks was lowered from 21% to 12%. Pbirth status indicates the statistical significance of the effect of term vs. preterm group on glucose metabolic rate. Pgas indicates the statistical significance of the effect of normoxic vs. hypoxic air on glucose metabolic rate. Pinteraction indicates the statistical significance of the interaction of term vs. preterm group and normoxic vs. hypoxic air on glucose metabolic rate.

Global metabolic measures were similar between term and preterm subjects throughout the study. Specifically, the serum glucose and lactate levels rose slightly: glucose rose by 5%/2% for term/preterm subjects (P_gas_ < 0.01, P_birth status_ = 0.62, P_interaction_ = 0.78) and lactate rose by 4%/11% for term/preterm subjects (P_gas_ = 0.11, P_birth status_ = 0.38, P_interaction_ = 0.32) (Figure 4).

### 3.3. PET Metabolic Rates of Glucose

**Figure 5:**
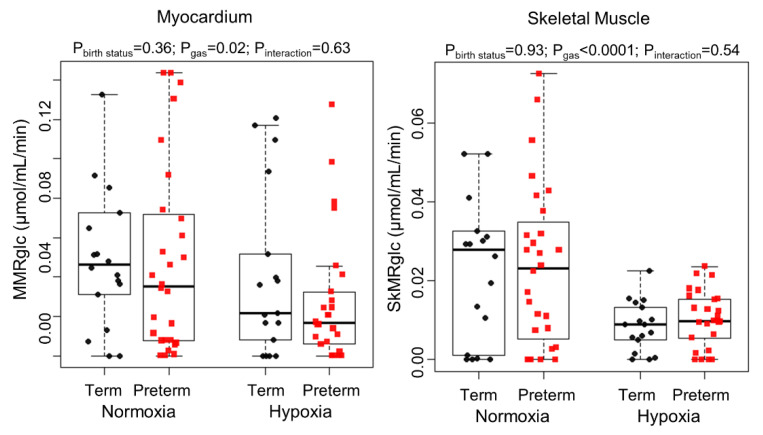
Dynamic PET measurements of the myocardial metabolic rate of glucose (MMRglc; left graph) and the skeletal muscle metabolic rate of glucose (SkMRglc; right graph). *p*-values listed at the top of the graphs are from linear mixed-effects models.

Myocardial and skeletal muscle glucose metabolism (MMRglc and SkMRglc) were similar at rest between term and preterm subjects (Figure 5). MMRglc and SkMRglc decreased during hypoxia exposure in both groups. Specifically, MMRglc decreased by 40% and 25% in preterm and term subjects, respectively, after hypoxia exposure (*p* = 0.02 for the effect of hypoxia). SkMRglc decreased by 52% and 64% in preterm and term subjects, respectively, after hypoxia exposure (*p* < 0.0001 for the effect of hypoxia). There were no significant differences in myocardial or skeletal muscle metabolic flexibility between the groups. 

**Figure 6:**
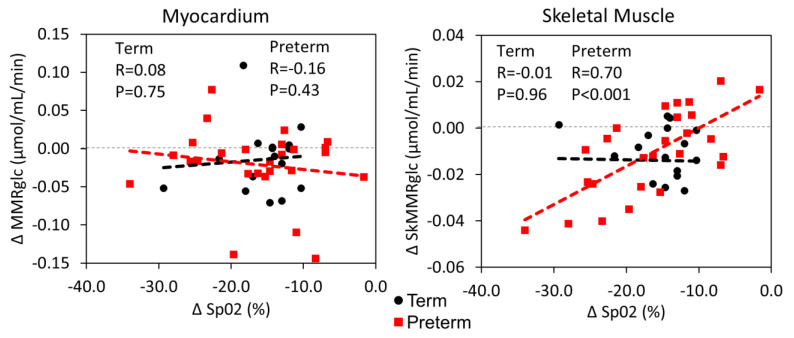
Correlation plots of the change in myocardial metabolic rate of glucose (ΔMMRglc; left graph) and the change in skeletal muscle metabolic rate of glucose (ΔSkMRglc; right graph) vs. the change in peripheral oxygen saturation (ΔSpO_2_) with hypoxia. Each graph also lists the Pearson correlation coefficient (R) and the *p*-value for the relationship between the variables from univariate linear regression in each group.

We investigated the relationship between the changes in MMRglc and SkMRglc and the degree of oxygen desaturation (ΔSpO_2_). The results are shown in Figure 6. While ΔSkMRglc was positively correlated with ΔSpO_2_ (R = 0.47, *p* = 0.001), ΔMMRglc was not significantly correlated with ΔSpO_2_ suggesting other factors were responsible for modulation of cardiac glucose metabolism.

## 4. Discussion

We used dynamic ^18^F-FDG PET imaging before and during a hypoxic gas challenge to measure cardiac glucose metabolism and the shift in glucose metabolism in response to hypoxia in young adults born premature and age-matched controls. We did not observe any differences in baseline cardiac glucose metabolism between preterm and term subjects. In response to hypoxia, both term and preterm subjects reduced glucose metabolism moderately in the myocardium and dramatically in skeletal muscle. There were no differences observed between groups in the metabolic response to hypoxia, either globally (serum glucose and lactate measures) or within the myocardium or skeletal muscle. Thus, we did not find evidence that changes in myocardial metabolism are present in the otherwise healthy preterm-born adult. However, whether subtle changes in myocardial metabolism may precede or predict heart failure in this population remains to be determined. 

Overall, our results demonstrate the capability of using simultaneous cardiac PET/MR to measure meaningful changes in cardiac function and metabolism with a rest-stress scenario within the same scanning protocol. The results for LV function and morphology are in line with the literature [4,6,7,8,29], demonstrating a smaller cardiac chamber size overall in those born preterm. However, MMRglc values were similar between healthy term and preterm-born adults. The median MMRglc values we measured during normoxia (roughly 0.04 μmol/mL/min) were 11 to 17 times lower than those reported in the literature after glucose loading (0.45–0.69 μmol/mL/min) [25,30,31] and 6 times lower than those reported after fasting (0.24 μmol/mL/min) [30]. We attribute the lower values we measured to the slow continuous FDG infusion in addition to the low-carbohydrate diet we asked our subjects to follow, which is known to reduce glucose uptake substantially even when compared to fasting alone [32,33]. This diet, in addition to fasting before the scan, is used clinically to suppress myocardial glucose utilization in healthy myocardium, offering high image contrast in disease states which elevate myocardial glucose utilization such as ischemia [34] and cardiac sarcoidosis [35,36]. We chose this diet to intentionally suppress myocardial glucose uptake in term-born subjects, hypothesizing that glucose metabolism would remain high in preterm-born subjects despite the diet, indicating altered metabolic activity. However, the results showed low glucose metabolism in both birth groups. 

While some previous studies found increases in cardiac glucose metabolism with hypoxia, we observed a decrease in cardiac glucose metabolism with hypoxia. There are two documented physiological phenomena that occur in myocytes during hypoxia exposure: reduced peripheral metabolism and a shift in metabolism from fatty acids to glucose metabolism. Hypoxia slows metabolism globally to reduce oxygen demand in part by lowering the activity of the electron transport chain through activation of the transcription factor hypoxia-inducible factor-1 [37]. In the myocardium, this phenomenon of reduced metabolic demand under hypoxic conditions, termed “hibernating myocardium”, is seen in patients with ischemic heart disease, and is associated with a shift in metabolism from fatty acid oxidation towards glycolysis [38]. One explanation for our finding of reduced glucose metabolism during hypoxic stress is that in our subjects the myocytes did not experience severe enough hypoxia to induce the shift towards glycolysis, while the phenomenon of globally reduced metabolism was realized, highlighted by the substantial reduction in SkMRglc and the increase in plasma glucose. Substantiating our findings, an increase in plasma glucose during acute hypoxia exposure has been observed in previous reports [17,32]. Our subjects did not become as hypoxic on average (SpO_2_ = 83%) as the 12 human subjects from the study of Chen et al. (SaO_2_ < 80%) or the 10 pigs from the study of Barton et al. (Sp0_2_ = 71%), which may explain our differential results. Additionally, these other studies had longer time periods between initial FDG infusion and image acquisition: Barton et al. imaged in normoxic conditions for 30 min then under hypoxic for 40 min, and Chen et al. waited 60 min after infusion to image. We were limited by total scanning duration, and this difference may have reduced our sensitivity to detect metabolic differences under normoxic conditions. 

While the extent of hypoxemia experienced likely played a role in causing the reduced glucose uptake in both cardiac and skeletal muscle in this study, another important mechanism may be a factor. Insulin action is markedly diminished during acute hypoxia [39], which is pertinent given that glucose uptake in striated muscle is insulin mediated [40,41,42]. Taken together, future studies looking at oxygen dose titration effects on glucose uptake into the heart combined with measurement of plasma insulin would help provide greater mechanistic insight into the individual contributions of oxygen content and insulin action on glucose uptake in the myocardium. The study by Lewandowski et al., which found greater insulin and total cholesterol levels in young adults born premature, highlights the utility of metabolic profile workup in this population [5]. Although the present study measured only fasting blood glucose and lactate, finding normal levels, a more sensitive marker of glucose metabolism such as glycated hemoglobin, insulin, or other systemic biochemical parameters could provide a more complete insight into metabolic abnormalities in survivors of preterm birth. The combination of specific localized metabolic measures from PET and general metabolic blood assays represents a promising direction for future work in this population.

Our hypothesis of cardiac metabolic differences in young adults born premature was not confirmed by our experiments. However, the preterm subjects in this study all had normal cardiac function, and thus it still remains to be seen whether such metabolic shifts may present later in the lifetime course and progression to dysfunction in some individuals. Furthermore, breathing hypoxic gas did not elicit a differential response in LV myocardial metabolism in young adults born premature relative to term-born controls. However, recent work suggests that the preterm heart may have contractile reserve (under hypoxic stress), particularly in the RV, [29] despite reduced volumetric reserve (under physiologic loading) [8,9]. As we were unable to capture the RV metabolic response to hypoxia due to the limitations of PET voxel size relative to RV wall thickness, whether there is altered RV metabolism remains to be determined. Moreover, a volume challenge (i.e., exercise) may have yielded a differential metabolic response in the preterm heart, despite the fact that the hypoxia challenge did not, and could be considered for future studies. Given the compensatory metabolic ability of the heart in the absence of strong secondary insults or comorbidities, a longitudinal study of cardiac function and metabolism would be ideal to chart the pathway towards dysfunction and failure in affected individuals. However, the present study, cross-sectional in nature, offers valuable initial insights into cardiac metabolism in survivors of preterm birth.

Our study had several limitations which might have reduced our ability to detect metabolic differences. A major limitation of this study is the use of only one tracer to assess myocardial metabolism. Myocardial metabolism in healthy subjects at rest draws 60–90% of its energy from fatty acid oxidation and 10–40% from glucose oxidation [43]. Due to the lack of locally available fatty acid tracers such as 11C-palmitate [39] or (18)F-fluoro-6-thia-heptadecanoic acid (18)F-FTHA [44,45,46], we were limited to measuring only glucose metabolism via (18)F-FDG. The increase in rate-pressure product suggests that hypoxia induced an increase in myocardial oxygen demand [47] and energy usage, but we did not measure an increase in glucose metabolism. We hypothesize that this increase in energy demand was met with an increase in fatty acid metabolism. We could not measure free fatty acid concentrations in the plasma (due to the lack of equipment to make that measurement with radioactive blood) or in the heart (due to the lack of access to tracers), so we were unable to assess this hypothesis in this study. Additionally, mitochondrial respiration data from this cohort showed elevated mitochondrial oxygen consumption at baseline in preterm subjects [48], yet baseline glucose metabolism did not differ between groups, reflecting either the influence of non-glucose energy sources, the lack of specificity in the mitochondrial measurement of a circulating peripheral blood cell, or limited sensitivity of the PET method. Future work using 11-C acetate would provide insight into mitochondrial oxidative metabolism within the heart specifically [49]. A second limitation is the only modest reduction in oxygen saturation experienced by this study’s participants, which may relate to the lack of an increase in myocardial glucose metabolism with hypoxia. Third, as previously mentioned, we measured glucose metabolism only in the LV myocardium due to the limited spatial resolution of PET imaging, and other imaging studies based on echocardiography and MRI suggest that the RV is more affected by preterm birth than the LV [4,5,6]. This is important considering previous work in the NLP cohort showed that adults born preterm exhibit increased mean pulmonary artery pressure during normoxia and hypoxia exposure [9]. Finally, the FDG signal in the myocardium was fairly low as previously discussed, and this low signal may have reduced the accuracy of our FDG kinetic model fits.

In conclusion, we used FDG PET to measure cardiac glucose metabolism and the shift in glucose metabolism in response to hypoxia in young adults born premature and age-matched controls. Despite inducing metabolic shifts in the myocardium and in the skeletal muscle, we did not observe any differences in glucose metabolism between groups. Future work should explore cardiac fatty acid metabolism in this population.

## Figures and Tables

**Table 1:** Baseline characteristics.

	Term	Preterm	*p*-Value
N	18	28	--
Male Sex (*n*, %)	8, 44%	8, 29%	0.27
Age (years)	25.4 (4.0)	25.8 (4.4)	0.75
Gestational age at birth (weeks)	39.8 (1.0)	29.2 (2.7)	<0.001
Birth weight (kg)	3.40 (0.57)	1.23 (0.42)	<0.001
BPD (*n*, %)	0, 0%	8, 29%	<0.001
PDA (*n*, %)	0, 0%	10, 36%	<0.001
Height (m)	1.73 (0.09)	1.66 (0.07)	0.01
Weight (kg)	70.0 (9.6)	71.1 (19.4)	0.81
Body surface area (m^2^)	1.83 (0.17)	1.80 (0.25)	0.65
Total physical activity (MET-mins./wk.)	2510 (2225)	4043 (5580)	0.18

Values shown are mean (standard deviation) unless otherwise noted. Abbreviations: N = number of subjects, BPD = bronchopulmonary dysplasia, PDA = patent ductus arteriosis, MET = metabolic equivalent. Boldface p-values signify statistical significance.

**Table 2:** Cardiac MRI measurements and vital signs.

	Term (N = 18)		Preterm (N = 28)		*p*-Values		
	Normoxia	Hypoxia	Normoxia	Hypoxia	Birth Status	Gas	Interaction
HR (bpm)	67 (13)	72 (13)	69 (11)	81 (15)	0.13	<0.0001	0.09
LV EDVi (mL/m^2^)	87 (16)	84 (16)	78 (10)	73 (12)	0.01	<0.0001	0.08
LV ESVi (mL/m^2^)	37 (9)	33 (9)	31 (6)	25 (6)	0.001	<0.0001	0.11
LV SVi (mL/m^2^)	50 (9)	51 (10)	47 (7)	48 (9)	0.25	0.49	0.59
LV EF (%)	57 (5)	61 (6)	61 (5)	66 (7)	0.01	<0.0001	0.28
CI (L/min/m^2^)	3.29 (0.78)	3.60 (0.61)	3.24 (0.57)	3.82 (0.78)	0.65	<0.001	0.23
SBP (mmHg)	123 (11)	126 (10)	125 (17)	124 (17)	0.87	0.13	0.12
DBP (mmHg)	71 (7)	74 (7)	74 (10)	73 (10)	0.64	0.21	0.12
RPP (mmHg/min)	7980 (1680)	9640 (1520)	8460 (1810)	10320 (1820)	0.22	<0.0001	0.30
Sp0_2_ (%)	98 (1)	85 (8)	98 (1)	81 (9)	0.56	<0.0001	0.50

Values shown are mean (standard deviation). Left ventricular volumes, ejection fraction, heart rate, and cardiac index were derived from cardiac MR images. Boldface p-values signify statistical significance. Abbreviations: LV = left ventricle, EDVi = end diastolic volume index, ESVi = end systolic volume index, SVi = stroke volume index, EF = ejection fraction, CI = cardiac index, SBP = systolic blood pressure, DBP = diastolic blood pressure, RPP = rate pressure product, SpO_2_ = peripheral oxygen saturation.

## Data Availability

The data that support the findings of this study are available on request from the corresponding author. The data are not publicly available due to privacy restrictions. The code used to perform the 3-compartment model fits has been made publicly available at https://github.com/pcorrado/Dynamic-Cardiac-FDG-Kinetic-Modeling (accessed on 21 March 2021).
